# Supplementary material for: Risk factors associated with poor pain outcomes following primary knee replacement surgery: Analysis of data from the clinical practice research datalink, hospital episode statistics and patient reported outcomes as part of the STAR research programme
Source: PLoS One. 2021 Dec 31;16(12):e0261850. doi: 10.1371/journal.pone.0261850 (PMC8719727; doi:10.1371/journal.pone.0261850)
Supplement: S1 Table — (DOCX) [file pone.0261850.s001.docx]

**S1 Table.** Imputation model checks with descriptive statistics and univariable model logistic regression results comparing complete case to imputed data.

| **Variable** | **Missing** | **Complete Case** | **Imputed** | **Regression** |  |
| --- | --- | --- | --- | --- | --- |
|  |  |  |  | **Complete Case** | **Imputed** |
| BMI | 352 (7.4%) |  |  |  |  |
| Normal |  | 650 (14.8%) | 712 (15.0%) | REF | REF |
| Overweight |  | 1692 (38.5%) | 1,835 (38.6%) | 1.60 (1.16, 2.21) | 1.55 (1.12, 2.15) |
| Obese class I |  | 1236 (28.1%) | 1,330 (28.0%) | 1.83 (1.31, 2.54) | 1.77 (1.28, 2.46) |
| Obese class II |  | 589 (13.4%) | 629 (13.2%) | 2.00 (1.39, 2.86) | 1.96 (1.37, 2.81) |
| Obese class III |  | 231 (5.3%) | 244 (5.1%) | 1.67 (1.04, 2.68) | 1.70 (1.06, 2.72) |
| Smoking | 81 (1.7%) |  |  |  |  |
| Ex |  | 1776 (38.0%) | 1,804 (38.0%) | 1.19 (0.99, 1.43) | 1.19 (0.99, 1.43) |
| No |  | 2598 (55.6%) | 2,646 (55.7%) | REF | REF |
| Yes |  | 295 (6.3%) | 300 (6.3%) | 2.71 (2.14, 3.43) | 2.72 (2.15, 3.45) |
| Drinking | 816 (17.2%) |  |  |  |  |
| Ex |  | 117 (3.0%) | 143 (3.0%) | 1.25 (0.77, 2.04) | 1.24 (0.77, 1.99) |
| No |  | 647 (16.5%) | 782 (16.5%) | REF | REF |
| Yes |  | 3170 (80.6%) | 3,825 (80.5%) | 0.84 (0.66, 1.07) | 0.82 (0.65, 1.04) |
| Quintile of Index of Multiple Deprivation (IMD) score | 5 (0.1%) |  |  |  |  |
| Least deprived ̶ 1 |  | 1185 (25.0%) | 1,186 (25.0%) | REF | REF |
| 2 |  | 1225 (25.8%) | 1,226 (25.8%) | 1.15 (0.89, 1.49) | 1.15 (0.89, 1.49) |
| 3 |  | 1055 (22.2%) | 1,056 (22.2%) | 1.16 (0.89, 1.51) | 1.16 (0.89, 1.51) |
| 4 |  | 789 (16.6%) | 790 (16.6%) | 1.44 (1.10, 1.88) | 1.44 (1.10, 1.88) |
| Most deprived ̶ 5 |  | 491 (10.4%) | 492 (10.4%) | 2.07 (1.58, 2.71) | 2.07 (1.58, 2.71) |
