# Supplementary material for: Risk factors associated with poor pain outcomes following primary knee replacement surgery: Analysis of data from the clinical practice research datalink, hospital episode statistics and patient reported outcomes as part of the STAR research programme
Source: PLoS One. 2021 Dec 31;16(12):e0261850. doi: 10.1371/journal.pone.0261850 (PMC8719727; doi:10.1371/journal.pone.0261850)
Supplement: S1 Fig — (DOCX) [file pone.0261850.s002.docx]

**S1 Figure** Receiver Operating Characteristic curve (ROC) curve for discriminatory ability of variables in the final model
