# Supplementary material for: Risk factors associated with poor pain outcomes following primary knee replacement surgery: Analysis of data from the clinical practice research datalink, hospital episode statistics and patient reported outcomes as part of the STAR research programme
Source: PLoS One. 2021 Dec 31;16(12):e0261850. doi: 10.1371/journal.pone.0261850 (PMC8719727; doi:10.1371/journal.pone.0261850)
Supplement: Attachment — Submitted filename: STAR PLOS ONE response to reviewers 2nd review 6Dec21 AJ.docx [file pone.0261850.s004.docx]

Professor Andrew Judge

Musculoskeletal Research Unit, Translational Health Sciences, Bristol Medical School,

University of Bristol, Learning and Research Building, Level 1, Southmead Hospital,

Bristol, BS10 5NB, UK

[Andrew.Judge@bristol.ac.uk](mailto:Andrew.Judge@bristol.ac.uk)

19 Oct 2021

Armando Almeida

Academic Editor

PLOS ONE

**RE: PONE-D-21-27576R1**

**Risk factors associated with poor pain outcomes following primary knee replacement surgery: analysis of data from the Clinical Practice Research Datalink, Hospital Episode Statistics and Patient Reported Outcomes as part of the STAR research programme**

Dear Dr Almeida,

Thank you for reviewing our manuscript at PLOS ONE as per your email dated 15 Oct 2021. We have considered these comments carefully, and our responses and amendments to the manuscript are reported below.

|  | **Reviewers' comments to the Author:** | **Response:** |
| --- | --- | --- |
|  | **Reviewer #1**: I thank the authors for their answers to my previous comments. I have some additional suggestions that are detailed below. | Thank you, we are very grateful for your comments. |
| 1. | INTRODUCTION  Page 3, first paragraph: “Many patients can expect to achieve reductions in knee pain and improvements in functional outcomes [3]. The percentage who experience ongoing pain is variable [4], with up to 20% experiencing knee pain that impacts their quality of life [5]. Patients who experience this kind of pain after surgery have not received the expected benefit and for some their pain is worse than it was before the operation [6, 7].” – In this paragraph, the time point at which these patients still experience pain is not clear. I can assume that it refers to chronic pain, but it should it should be stated. | Thank you for your comments we agree and have amended these sentences.  *Many patients can expect to achieve reductions in knee pain and improvements in functional outcomes following surgery [3]. The percentage who experience ongoing chronic knee pain post-surgery is variable [4], with up to 20% experiencing knee pain that impacts their quality of life after 3 months post-op [5]. Patients who experience this kind of pain after surgery have not received the expected benefit and for some their pain is worse than it was before the operation [6, 7].*  [Introduction, paragraph 2] |
| 2. | Page 3, last paragraph: “There is limited research focusing solely on pain status”. In fact, there is a large body of research focusing on acute and chronic pain after surgery, including systematic reviews. I do not think that this sentence is an accurate depiction of the state of the art. | We agree and have amended the sentence, to clarify our meaning.  *Although previous research has explored predictors of outcomes of knee replacement [8], most studies have focused on total scores encompassing several domains (e.g. pain, stiffness and function) and fewer studies have focussed solely on pain status [7].*  [Introduction, paragraph 2] |
| 3. | Page 3, last paragraph: “This is important given that up to 20% of patients will have long-term pain after surgery [5].” – This information is already stated in the previous paragraph. | We agree and have removed the sentence.  [Introduction, paragraph 2] |
| 4. | Page 4: Please consider if the objective would be improved by stating that the aim was to “identify pre and postoperative risk factors”, since the authors present this strategy as a novelty. | We agree and have added this statement.  *The aim of this study is to identify pre- and post-operative risk factors for whether or not a patient has a poor pain outcome after knee replacement surgery…*  [Introduction, paragraph 3] |
| 5. | RESULTS  Table 1: I appreciate the clarifications made by the authors concerning Table 1. However, it is my opinion that the information in the table is not very intuitive to understand. One suggestion would be to change the heading of the third column to “Proportion of patients with poor pain response with and without each risk factor”. And then change the “Yes” and “No” to “With” and “Without”. | We agree and have amended the table headings. ***Proportion of patients with poor pain response with and without each risk factor*** \|; ***Without*** \| ***With*** [Table 1] |
| 6. | Also, please consider if using the term “risk factor” is adequate in this context. Only the statistical analyses tell us if each characteristic is a risk factor or not (and not the descriptive data). | We agree and have clarified our wording for the descriptive data.  *The aim of this study is to identify pre- and post-operative risk factors for whether or not a patient has a poor pain outcome after knee replacement surgery, by analysing a wide range of potential factors from the UK Clinical Practice Research Datalink (CPRD) primary care GOLD database linked to English Hospital Episode Statistics (HES) hospital admissions and to Patient Reported Outcomes Measures (PROMs) data.*  [Introduction, paragraph 3]  ***Table 1. Descriptive statistics describing the total number of patients with each potential risk factor, and the proportion of patients with a poor pain outcome, according to whether or not they have the factor***  [Table 1] |
| 7. | Since this table only presents descriptive information, do the authors believe that it is accurate to make claims about risk factors based on its information? Probably the most accurate way of stating the results would be, for example “The highest proportion of patients with poor pain outcomes were in the group of current smokers, males, people living in the most deprived areas and those with inflammatory arthritis.” | We agree and have amended this sentence.  *The highest proportion of patients with poor pain outcomes were in the group of current smokers, males, people living in the most deprived areas and those with inflammatory arthritis (Table 1).*  [Results, paragraph 3] |
| 8. | The rationale to select reference categories should be clear in the statistical analyses section, even if it is usual practice. | We agree and have added the following sentence.  *As our dataset is large, we selected the lowest category for each variable as the reference category.*  [Statistical analysis, paragraph 2] |
| 9. | The information provided by the authors in the response letter concerning the analysis of strength of association should be stated in the statistical analysis section (We are examining the strengths of association and not arbitrary measures of statistical significance with cut offs of for example p<0.05 or the related concept of whether the confidence interval includes the null value.) | We agree and have added this sentence.  *We examine the strength of associations and not arbitrary measures of statistical significance with cut offs of for example p<0.05 or the related concept of whether the confidence interval includes the null value.*  [Statistical analysis, paragraph 2] |
|  | **Reviewer #2:** Comments addressed satisfactorily | Thank you. |
|  | **Reviewer #3:** The authors have done well to address all the reviewer comments. Some reanalysis has been undertaken and substantial amendments to the manuscript have been made | Thank you. |

We hope our responses are satisfactory, but please do not hesitate to contact us if more information is required.

Yours sincerely

Professor Andy Judge
